# Successful Living Kidney Donor Transplantation: A Case Report

**DOI:** 10.1155/crit/2822007

**Published:** 2025-02-18

**Authors:** Meredith Wells, Keith Melancon, Robert Young, Adam Greenwood, Joseph Manley, Pablo Serrano Rodriguez

**Affiliations:** ^1^School of Medicine and Health Sciences, George Washington University, Washington, DC, USA; ^2^Department of Surgery, George Washington University Hospital, Washington, DC, USA

## Abstract

This case report describes the successful kidney donation of a 68-year-old female to a 56-year-old female recipient with end-stage renal disease (ESRD). The donor in this case presented with an extensive abdominal surgical history, which had resulted in an incidental injury to the right ureter and a subsequent nephrostomy tube placement. After establishing the low likelihood of reconstruction of the damaged ureter, she then initiated kidney donor evaluation after electing to undergo a nephrectomy. This case report highlights the meticulous evaluation and preparation of the donor under particular circumstances, the surgical techniques employed, and the positive outcomes for both the donor and the recipient.

## 1. Introduction

Iatrogenic ureteral injury is a complication of abdominal surgery with potentially devastating consequences. It is a feared complication of gynecological surgeries as well as a wide variety of other abdominal surgeries, from vascular surgeries to small and large bowel resections. The majority of ureteral injuries occur in the distal ureter [1]. Options to manage iatrogenic damage to the distal ureter in the long term include Boari tubularized bladder flap, psoas hitch, transureteroureterostomy, or renal autotransplantation [1]. In this case report, we present another option: living kidney donation.

According to the most recent Organ Procurement and Transplantation Network (OPTN) data, there are currently 96,056 people on the kidney transplant waiting list in the United States [2]. In the year 2022, there were a total of 20,093 kidney donations, with just 5865 of them being living kidney donations [3]. With the high demand for kidney donation in combination with the shortage of living kidney donors, presenting additional options for living kidney donation would provide potentially life-saving interventions for those with end-stage renal disease (ESRD). This case report presents a successful living kidney transplant procedure in the context of iatrogenic ureteral injury, emphasizing its feasibility and the benefits of this approach.

## 2. Case Report

### 2.1. Donor Evaluation

Our donor is a 68-year-old female with a past medical history of hypothyroidism and an extensive past surgical history that included a hysterectomy, small bowel resection with adhesiolysis, partial right colectomy, an incisional hernia repair, and a subsequent small bowel obstruction requiring resection with an inadvertent injury to the right ureter.

In December 2020, she presented to the emergency department with a 2-day history of abdominal pain, nausea, and bilious emesis concerning a small bowel obstruction. She was taken for a diagnostic laparoscopy followed by resection on December 18, 2020. She was left with an open abdomen and had a second look with bowel anastomosis the following day. On the 27th, fever and leukocytosis prompted an infectious workup. A subsequent CT showed right lower quadrant retroperitoneal collection concerning an abscess or urinoma measuring up to 16.7 cm. A ureterogram demonstrated damage to the distal ureter approximately 7 cm proximal to the ureteral orifice. Reconstruction was attempted; however, it was unsuccessful. The following day, a nephrostomy tube was placed.

**Figure 1:**
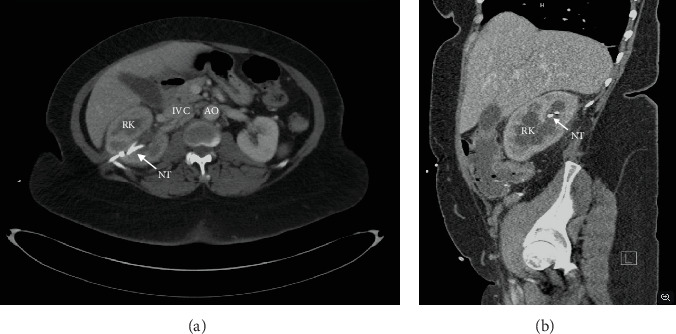
(a) Donor evaluation axial abdomen/pelvis CT with contrast depicting moderate right hydroureteronephrosis of the right kidney. (b) Sagittal CT abdomen/pelvis with contrast depicting moderate right hydroureteronephrosis with a transition point in the right mid ureter, compatible with stricture. The right percutaneous nephrostomy tube (NT) is able to be visualized in both planes, terminating within a right upper pole calyx. There is a 1.3 cm indeterminate hypodense lesion in the left upper pole, stable in size compared to the prior CT on 02/16/2023. IVC, inferior vena cava; AO, aorta; RK, right kidney; NT, nephrostomy tube.

Prior to proceeding with the donation, the patient underwent a thorough evaluation by the urology team. Alternative procedures were explored but deemed unsuitable due to her surgical history. Both ureteroureterostomy and nephrectomy with kidney autotransplantation were weighed alongside the risks of further surgery in the context of the extensive adhesions. Due to her extensive surgical history, surgical risks associated with adhesions, such as prolonged operative times required for careful dissection of the adhesions, increased risk of injury to abdominal organs due to the obscured surgical field, and increased risk of future intestinal obstruction, were outlined to the patient and carefully considered in regards to future surgical options [4]. Finally, after evaluating the risks and benefits, the patient expressed a preference for nephrectomy. Her renal function was deemed adequate based on nuclear medicine testing. She presented to our clinic for an extensive donor evaluation and was deemed an appropriate candidate with no contraindications. Assessment of preoperative imaging, as shown in Figure 1, demonstrated appropriate renal morphology and vascularity for donation. Ultimately, the patient's preference to avoid a longer hospital stay with a higher rate of complications, along with the medical team's assessment of adequate kidney function in her remaining kidney, led us to pursue nephrectomy as the least invasive and most viable option. Using the ESRD Risk Tool for Kidney Donor Candidates, we calculated this patient's lifetime risk of developing ESRD postdonation to be less than 1% [5]. This low risk is attributed to her favorable health profile, including the absence of hypertension (HTN) and diabetes, a healthy body mass index, and adequate baseline kidney function. The absence of these risk factors significantly reduces her likelihood of kidney disease progression compared to individuals with comorbidities or suboptimal health. Although kidney donation slightly increases the relative risk of ESRD due to the loss of one kidney, her absolute risk remains minimal given her optimal health status. The patient met all institutional medical, surgical, and psychosocial criteria and then underwent an open right nephrectomy without complications.

### 2.2. Recipient Evaluation

We initially sought a recipient to initiate a kidney donation chain, as is commonly practiced with altruistic kidney donations at our institution. However, given the anticipated challenges of short vascular structures and ureters from the donor, we prioritized identifying a smaller recipient. This approach was aimed at optimizing the compatibility of the donor kidney and minimizing the risk of surgical complications for the recipient.

Our recipient is a 56-year-old woman with a past medical history of diabetes and HTN-induced ESRD diagnosed in 2019. She presented to the clinic for transplant evaluation and was subsequently listed in August 2020. She underwent a thorough evaluation to assess her suitability as a kidney transplant recipient. Her medical and surgical history, psychosocial factors, and immunological compatibility were considered. Her serum creatinine level was 6.8 mg/dL preoperatively, and she was dependent on dialysis three times per week.

### 2.3. Surgical Procedure

The procedure was initiated with a laparoscopic approach with a standard right donor nephrectomy technique. The donor was placed in a modified lateral decubitus position, and a laparoscopic 12 mm port at the umbilicus was inserted to be used as a camera port. This allowed for visualization of anticipated adhesions due to the extensive past surgical history in the donor and preoperative assessments. The port placement allowed for visual confirmation of the significant adhesions that precluded a laparoscopic approach, and the procedure was converted to open laparotomy without complication. Two renal arteries and one vein were taken with a vascular stapler. Subsequently, the kidney was flushed and handed to the recipient team. A ventral hernia repair was performed before closing. The transplantation procedure was completed without complication.

## 3. Results

### 3.1. Postoperative Course

Our recipient received institution-standard immunosuppressive induction. She had an episode of antibody-mediated rejection that was treated with plasma exchange and intravenous immunoglobulin. Creatinine was stable at 2.3 mg/dL upon discharge on postoperative Day 17. Our donor had an uncomplicated postoperative course and was discharged on postoperative Day 4. She was seen in the clinic with adequate creatinine clearance and excellent quality of life.

## 4. Discussion

Organ scarcity is a significant contributor to the mortality rate of patients with ESRD. Living kidney donation has provided a desperately needed expansion to the pool of available organs, allowing for more lifesaving kidney donations. Living kidney donors may present with differing risk factors; however, with the correct education and management, risks can be minimized, resulting in successful donations [6]. This case report underscores the success of living kidney donor transplantation as an option for patients who may have experienced iatrogenic ureteral damage from previous abdominal surgery. Though considered a more high-risk donor, after thorough evaluation and education to all parties, the donation was successful. We present this as an example that adherence to the pre-evaluation guidance protocol from the OPTN ensures that high-risk donors can be appropriately educated to make an informed decision.

Autotransplantation, while a valuable technique in certain clinical scenarios, is not without its risks. Studies indicate that while autotransplantation can be successful in cases like restoring the functionality of essential tissues or organs, the procedure carries significant risks and variable long-term outcomes. One study highlighted those complications, such as graft failure, infection, and the potential need for reoperation, which can occur, casting doubt on the procedure's benign nature [7]. Additionally, long-term follow-up data show that the success of autotransplantation can diminish over time, with some patients experiencing a decline in function or the development of secondary complications, suggesting that careful consideration and patient selection are crucial when opting for this approach.

This case emphasizes the importance of donor and recipient evaluation, surgical technique, and postoperative care with this unique presentation. It is important to encourage donation from those with atypical presentations such as this in order to increase the number of available donor organs [5]. Living kidney donation offers advantages such as shorter wait times and potentially better outcomes for the recipient, while donors can lead healthy lives with one kidney [8]. Living kidney donor transplantation is a viable and successful treatment option for patients with ESRD. This case report highlights the positive outcomes for both the living donor and the recipient, showcasing the importance of careful evaluation and surgical precision in achieving favorable results.

## Data Availability

Data sharing is not applicable to this article as no datasets were generated or analysed during the current study. All relevant information is included within the manuscript.
